# Developing an interpretable machine learning model for predicting COVID-19 patients deteriorating prior to intensive care unit admission using laboratory markers

**DOI:** 10.1016/j.heliyon.2023.e22878

**Published:** 2023-11-28

**Authors:** A. Reina-Reina, J.M. Barrera, A. Maté, J.C. Trujillo, B. Valdivieso, María-Eugenia Gas

**Affiliations:** a Lucentia Research. Department of Software and Computing System, University of Alicante, Carretera San Vicente del Raspeig s/n, 03690, Alicante, Spain; b Lucentia Lab, Av. Pintor Pérez Gil, 16, 03540, Alicante, Spain; c The University and Polytechnic La Fe Hospital of Valencia, Avenida Fernando Abril Martorell, 106 Torre H 1st floor, 46026, Valencia, Spain; d The Medical Research Institute of Hospital La Fe, Avenida Fernando Abril Martorell, 106 Torre F 7th floor, 46026, Valencia, Spain

**Keywords:** Machine learning, Covid-19, Laboratory markers, Precision medicine

## Abstract

Coronavirus disease (COVID-19) remains a significant global health challenge, prompting a transition from emergency response to comprehensive management strategies. Furthermore, the emergence of new variants of concern, such as BA.2.286, underscores the need for early detection and response to new variants, which continues to be a crucial strategy for mitigating the impact of COVID-19, especially among the vulnerable population. This study aims to anticipate patients requiring intensive care or facing elevated mortality risk throughout their COVID-19 infection while also identifying laboratory predictive markers for early diagnosis of patients. Therefore, haematological, biochemical, and demographic variables were retrospectively evaluated in 8,844 blood samples obtained from 2,935 patients before intensive care unit admission using an interpretable machine learning model. Feature selection techniques were applied using precision-recall measures to address data imbalance and evaluate the suitability of the different variables. The model was trained using stratified cross-validation with k=5 and internally validated, achieving an accuracy of 77.27%, sensitivity of 78.55%, and area under the receiver operating characteristic (AUC) of 0.85; successfully identifying patients at increased risk of severe progression. From a medical perspective, the most important features of the progression or severity of patients with COVID-19 were lactate dehydrogenase, age, red blood cell distribution standard deviation, neutrophils, and platelets, which align with findings from several prior investigations. In light of these insights, diagnostic processes can be significantly expedited through the use of laboratory tests, with a greater focus on key indicators. This strategic approach not only improves diagnostic efficiency but also extends its reach to a broader spectrum of patients. In addition, it allows healthcare professionals to take early preventive measures for those most at risk of adverse outcomes, thereby optimising patient care and prognosis.

## 1 Introduction

After the COVID-19 pandemic, the World Health Organization and the International Health Regulations Emergency Committee (2005) articulated the need for a transition from emergency response activities to the long-term management of COVID-19, alongside other infectious diseases [1]. Nonetheless, they also acknowledged the persisting uncertainties resulting from the potential evolution of the virus [1]. Indeed, three months later, the variants of concern, BA.2.86, were identified [2]. In this context, the imperative to promptly detect and respond to these variants remains a crucial strategy to mitigate the impact of COVID-19, particularly for vulnerable populations, with the overarching goal of advancing global health and well-being [3], [4], [5].

In order to mitigate COVID-19, recent works have shown the potential of artificial intelligence (AI) predictive models to improve the quality and accuracy of diagnoses [6], [7], [8], [9], [10], mortality [11], [12], [13], [14], [15], treatment [16], [17], risk stratification [18], hospital readmission risk [19], prognosis [20], [21], [22], drug repurposing [23] and development [24], management and reducing costs [25], or in the implementation of a personalised precision medicine [26] to address COVID-19 disease. Additionally, several studies have highlighted the association between COVID-19 and multi-organ dysfunction, especially in the most severely COVID-19-affected patients [27], [28]. Neurological [29], [30], cardiovascular [31] and respiratory disorders [32] are all mainly featured in COVID-19. Therefore, the development of AI-based tools capable of identifying at-risk patients is necessary to transform the current healthcare systems into more personalised, and proactive models of disease management [33].

More specifically, AI research on COVID-19 (patient deterioration) has been based on image analysis, comorbidities and laboratory test results. AI-based applications in medical imaging [34], [35] have revealed important details related to the development of severe respiratory infectious diseases. Lung chest X-ray image analysis has been successfully used to classify patients severely affected by COVID-19 [17], [36]. Govardhan et al. [17] developed a two-step AI-based model to detect COVID-19 using X-ray images so that adequate treatment could be given. In the first stage, a predictive model differentiates viral-induced pneumonia from bacteria-induced pneumonia and normal/healthy people. In the second stage, the application of a predictive model allows the detection of the presence of pneumonia caused by the COVID-19 virus from the pneumonia induced by other viruses. Wenli Cai et al. [20] analysed CT images to build an AI-based model aimed at the assessment of COVID-19 disease severity and the prediction of clinical outcomes. Zakariaee et al. show that the odds of mortality for COVID-19 patients could be accurately predicted using an optimal chest CT severity score in visual scoring of lung involvement [15]. Despite these positive contributions to COVID-19 clinical research, several works have highlighted some limitations of studies based on medical image analysis. Firstly, in early-stage disease or in patients with mild symptoms, chest images may be normal [37]. Secondly, analysis of chest X-ray and computed tomography scan (CT-Scan) in COVID-19-infected patients can be expensive and time consuming due to the need to strictly adhere to infection control protocols designed to minimise the risk of transmission and protect healthcare workers [38]. Therefore, there is a need to develop more cost-effective and less resource-intensive strategies that can be applied at earlier stages of the disease, based on the analysis of other clinical records stored in the medical record, is necessary.

Analysis of pre-existing comorbidities has been proven to be valuable in predicting COVID-19 outcomes [8], [39], [40], diagnosis [10], even in survival analysis on censored data [41]. However, their applicability might be hampered due to the fact that: i) comorbidities prevalence varies along countries and regions [42] due to socio-political factors, health equity issues, and environmental threats [43], [44]; ii) there are discrepancies and variability in data collection systems as well as in the version of international classification of diseases (ICD) used across different institutions and countries, hindering meaningful comparison or introducing research bias [42], [45] by producing skewed results as a consequence of the relationship between some comorbidities and death rates [46]; iii) inclusion of pre-existing comorbidities analysis is required [37]; iv) Achieving a high level of digital transformation maturity is necessary [42], [47] to ensure models robustness and usability.

Compared to comorbidities models, the application of AI models based on blood laboratory samples does not require the analysis and processing of historical patient data. Furthermore, blood laboratory samples can be used to reduce the pressure on COVID-19 intensive care units and to detect at admission or in hospital severely and mildly infected COVID-19 patients [22]. In this sense, several works highlight their importance and effectiveness in the diagnosis, prognosis, mortality of COVID-19 [9], [12], [13], [14], [19], [21], [22], [48], [49], [50]. Huyut et al. [21] show a LogNNet Neural Network to assess the diagnosis and prognosis of COVID-19 disease using routine blood values. Mertoglu et al. [48] highlighted significant changes in routine blood tests between the intensive care unit (ICU) and non-ICU patients. Shanbehzadeh et al. [14] show the importance of absolute count value of neutrophil and lymphocyte to predict COVID-19 mortality in-Hospital. In fact, Afrash et al. also pointed lymphocytes on discharge as major risk factors for hospital readmission [19]. Similarly, authors in [49] identified routine parameters and some biomarkers as predictors of COVID-19 diagnosis and prognosis. Additionally, they determined the lethal-risk levels of procalcitonin and ferritin [12], underscoring the feasibility of utilising biomarkers as reliable indicators for managing COVID-19 disease progression. It is worth mentioning that laboratory markers are safe, easy to measure and have an acceptable cost (including those of the follow-up tests) [9], [51]. Nevertheless, the lack of information related to blood collection times in published studies makes predictive model replication and validation difficult [52], [53], [54]. Furthermore, some works developed to date base their conclusions on analyses of relatively small cohorts which may lead to data bias, as reported by Malik et al. [55] in their systematic review and meta-analysis. Hence, studies with larger cohorts of patients are needed to provide better robustness to the models.

To overcome above-mentioned limitations, the aim of this study is to anticipate the need for ICU care or the potential mortality risk among individuals throughout their COVID-19 infection. Additionally, we analyse demographic biochemical and haematological registries routinely collected across primary and secondary care that can serve as predictive indicators for the early identification of patient deterioration. With these objectives, we train a machine learning (ML) model, based on the analysis of a large cohort of data, that aims to identify vulnerable patients at risk of poor outcomes, enabling anticipation rather than a reactive response to severe disease progression.

The proposed model builds on the results of an earlier study [39], which allows us to develop an AI-based predictive model aimed at the identification of those patients at higher risk of dying in the event of COVID-19 infection based on demographic factors and comorbidities. In this study, we go further by analysing the impact of biochemical and haematology parameters, obtained from routine blood tests, on the model's performance. As a result of this analysis, the developed predictive model: i) is able to identify critical patients from blood tests performed 3.6 days post COVID-19-positive along with the rest of relevant variables; ii) does not depend on the analysis of pre-existing clinical data; iii) has increased robustness compared to the previous one; iv) used routinely collected electronic health record data in hospital settings; and v) included a feature reduction step in the analysis requiring lower input variables.

Finally, the article is divided into the following major sections: (1) the materials and method section where we describe the different steps performed on the data, inclusion and exclusion criteria, and other considerations. (2) The results sections describe the study cohort from the laboratory markers and present the results obtained, feature importance and prediction explanation. (3) The discussion section covers the different solutions presented depending on the type of variable used, and the advantages and limitations of our approach. Finally, (4) the conclusion section summarises the main contributions of the work presented.

## 2 Materials and method

### 2.1 Study design and setting

This retrospective study collects data from individuals undergoing a COVID-19 test at the Department of Health La Fe in Valencia (Spain) between 27 February 2020 to 15 April 2021. The aim of the study is to predict which patients will potentially require ICU care or may die during the course of COVID-19 infection (severe patients) and to identify predictive laboratory factors for early diagnosis of patientś severity.

The eligibility criteria were unvaccinated individuals infected with COVID-19, assigned to the University and Polytechnic La Fe Hospital, and whose infection was confirmed by Reverse Transcription Quantitative Polymerase Chain Reaction (RT-qPCR) tests and for which blood tests were available during their COVID-19 infection. No age limit was required. Vaccinated individuals were excluded because they represented a very small population at the time of the study and could introduce bias since vaccinated individuals were likely to differ from those who were unvaccinated.

**Figure 1:**
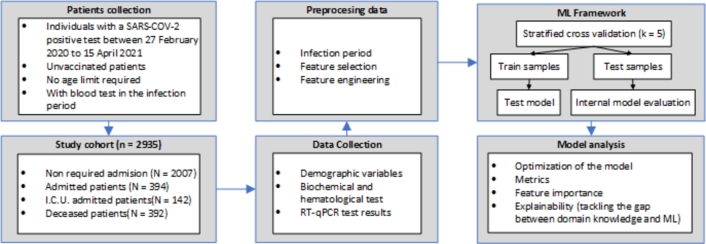
Flow chart of the study. ML stand for Machine Learning.

The study cohort comprised 2,935 patients, with 2,007 (68.38%) being outpatients, 394 (13.42%) being admitted to the hospital, and 142 (4.84%) being admitted to the ICU, making a total of 2,543 that survived (86.65%), while 392 (13.35%) died. In the cohort under study, we included data collected from different settings (hospital and ICU admission along with primary care). These data include demographic variables (age and sex), and biochemical and haematological records registered during the infection period of each patient. In Fig. 1 we can see the flow chart of the study. Furthermore, Table 1 shows the descriptive characteristics of the study population prior to the infection. More specifically, it describes the diagnoses and procedures recorded in the patient's medical history one month before infection, or those that were identified as chronic conditions prior to the infection.

**Table 1:** Diagnoses and procedures recorded in the patient's medical history one month prior to infection, or those that were identified as chronic conditions prior to the infection. Diagnoses and procedures descriptions are provided in international classification of diseases, ninth revision, clinical modification (ICD-9-CM) format [56].

	Non Severe (n = 2401)				Severe (n = 534)				All patients	
	Male (n =1206)		Female (n = 1195)		Male (n = 295)		Female (n= 239)		(n = 2935)	
Diagnoses and procedures	n	%	n	%	n	%	n	%	n	%

Unspecified essential hypertension	586	48.59%	568	47.53%	194	65.76%	176	73.64%	1524	51.93%
Other and unspecified hyperlipidemia	419	34.74%	412	34.48%	131	44.41%	115	48.12%	1077	36.70%
Diabetes mellitus without mention of complication. type ii or unspecified type not stated as uncontrolled	334	27.69%	268	22.43%	97	32.88%	80	33.47%	779	26.54%
Anxiety state unspecified	170	14.10%	378	31.63%	29	9.83%	66	27.62%	643	21.91%
Unspecified vitamin D deficiency	152	12.60%	336	28.12%	29	9.83%	45	18.83%	562	19.15%
Obesity unspecified	216	17.91%	241	20.17%	40	13.56%	55	23.01%	552	18.81%
Contact or exposure to other viral diseases	171	14.18%	201	16.82%	24	8.14%	24	10.04%	420	14.31%
Urinary incontinence unspecified	82	6.80%	196	16.40%	46	15.59%	77	32.22%	401	13.66%
Chronic kidney disease. unspecified	128	10.61%	138	11.55%	63	21.36%	44	18.41%	373	12.71%
Osteoarthrosis unspecified whether generalized or localized involving unspecified site	82	6.80%	201	16.82%	28	9.49%	55	23.01%	366	12.47%
Hypertrophy (benign) of prostate without urinary obstruction and other lower urinary tract symptoms (LUTS)	250	20.73%	0	0.00%	87	29.49%	0	0.00%	337	11.48%
Mixed hyperlipidemia	140	11.61%	137	11.46%	37	12.54%	25	10.46%	339	11.55%
Atrial fibrillation	104	8.62%	96	8.03%	72	24.41%	53	22.18%	325	11.07%
Unspecified acquired hypothyroidism	52	4.31%	199	16.65%	16	5.42%	40	16.74%	307	10.46%
Unspecified cataract	123	10.20%	108	9.04%	28	9.49%	29	12.13%	288	9.81%
Absence of chronic comorbidities or comorbidities during the month preceding infection	146	12.11%	102	8.54%	7	2.37%	9	3.77%	264	8.99%

Finally, all methods carried out in this study were implemented in Python 3.8. Pandas library [57] was utilised to process and operate through raw data. The models were developed and evaluated using the scikit-learn library [58]. Graphics and visualisations for the study were created using the Matplotlib [59] and Seaborn [60] libraries. For Pearson correlation analysis and obtaining the standard error and confidence interval of logistic regression (LR) coefficients, the Scipy [61] and Statsmodels [62] libraries were utilised. Additionally, Anaconda [63] was used for package management and deployment.

### 2.2 Data preprocessing

In this section, we will provide further details concerning the processing of the raw data from the collected data. Firstly, we determine the infection period by analysing RT-qPCR and serological tests. Furthermore, aspects of feature engineering and data normalisation will be explored to optimise the performance of our ML models.

#### 2.2.1 Traceability

During the patient's infectious period, we analysed 74,239 RT-qPCR and serological tests in order to identify early predictors of ICU admission or mortality. Patients whose period of infection could not be determined were not included in the study. The study period was understood as the elapsed time from the first positive RT-qPCR test results and the first immunoglobulin G seroconversion detected or epidemiological discharge. This allows us to narrow the patient's infectious period and not consider post-sequelae that may appear after the patient has overcome the infection. Finally, time windows shorter than 5 days were discarded.

#### 2.2.2 Feature engineering

In order to improve ML algorithms' performance domain, knowledge was used to select and/or transform variables from raw data. Gender and age-specific reference intervals provided by The University and Polytechnic La Fe Hospital of Valencia were used to analyse the biochemical and haematology analytics.

#### 2.2.3 Data normalization

Before training the ML algorithms, several data normalisation methods were used to standardise the scale of input features, ensuring uniformity and preventing features with larger magnitudes from dominating the model's learning process [64]. Therefore, MaxAbsScaler, Robust, Quant-Normal, quant-Uniform and Power Transform-yeo Jhonson were empirically evaluated in terms of numerical features. Similarly, one hot encoding technique was used to normalise the categorical variables such as sex.

### 2.3 Feature selection

**Figure 2:**
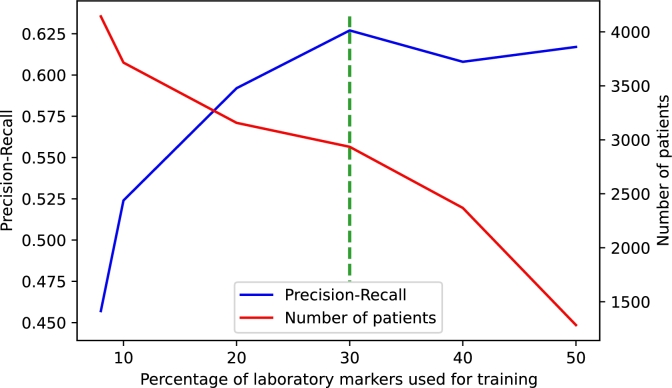
The diagram illustrates feature selection based on the availability of laboratory markers in patients. The left Y-axis depicts the logistic regression value measured by precision-recall. The right Y-axis depicts the number of patients frequency. The X-axis depicts the percentage of laboratory markers selected for training algorithms, ordered from highest to lowest availability. The dashed green line represents the chosen threshold. A percentage of 10% corresponds to approximately 13 different laboratory markers.

**Figure 3:**
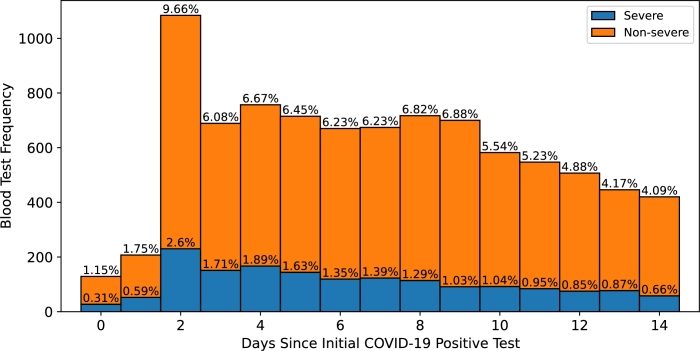
Temporal distribution of blood test collected through patients' infection period and prior to admission. The horizontal axis shows the days until the sample is collected from each patient's first positive test. Day 0 was defined as the one in which the initial COVID-19 positive test was collected. The vertical axis shows the stacked bars with the frequency and percentage of blood tests performed for all patients, categorized as severe and non-severe.

A total of 127 different laboratory markers were collected throughout the patient's infection. Therefore, the number of input variables was reduced by applying filter feature selection methods [65], [66] with the aim of maximising the number of patients included in the study while minimising the loss of informative laboratory markers. To construct the different datasets by the percentage of available laboratory markers, we weighted the number of patients according to the biochemical and haematological parameters available for the infection period from highest to lowest availability. Precision-Recall (also known as average precision) with an LR algorithm was used to evaluate the performance of feature selection on unbalanced datasets [67], [68]. In Fig. 2 we can find the diagram constructed to evaluate the results of the algorithms and the number of patients for different sets of laboratory markers availability. On the left side, poor results are obtained, probably because the number of laboratory markers is not sufficiently representative, or in other words, they do not have enough predictive weight. On the right side of the graph, we did not consider the results when selecting more than 50% of the different laboratory markers available because the number of patients who had all laboratory markers available dropped considerably. Finally, we see that the optimal case is found by selecting 30% of the available laboratory markers. It is key to note that laboratory markers collected after ICU admission were discarded. Fig. 3 shows all blood tests collected prior to admission and included in the study.

#### 2.3.1 Correlation analysis

**Figure 4:**
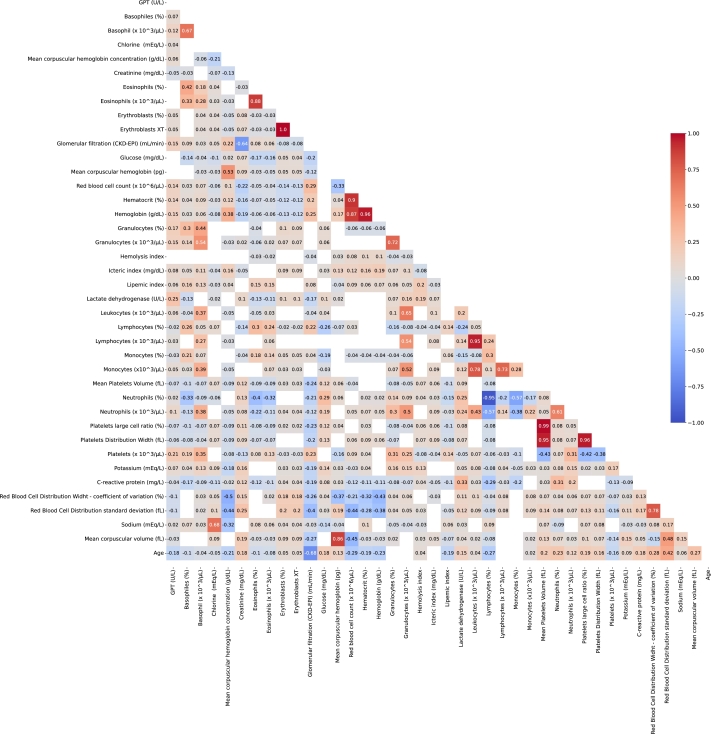
Correlation coefficient matrix heatmap of the variables included in the study. The value is shown if the value of the correlation has a p-value < 0.05. On the contrary, the cell is blank. Red colours indicate a positive correlation whereas blue colours indicate a negative correlation. Strong colours refer to stronger correlations close to 1 or -1. More specifically, stronger positive correlation is shown as redder and bluer respectively.

Moreover, Pearson correlation analysis was initially applied. Then, low-correlated variables were included in the LR model in order to avoid multicollinearity problems [69]. Variables with a high correlation (greater than 0.70) and a p-value of 0.01 were excluded. In Fig. 4 it is shown the correlation between: Erythroblasts XT and erythroblasts (%) (r=1.00), platelets large cell ratio (%) and mean platelets volume (fL) (r=0.99), hemoglobin (g/dL) and hematocrit (%)(r=0.96), platelets large cell ratio (%) and platelets distribution width (fL) (r=0.96), lymphocytes ($x10^{3}/\;\mu \text{L}$) and leukocytes ($x10^{3}/\;\mu \text{L}$) (r=0.95), mean platelets volume (fL) and platelets distribution width (fL) (r=0.95), red blood cell count ($x10^{6}/\;\mu \text{L}$) and hematocrit (%)(r=0.90), eosinophils (%) and eosinophils ($x10^{3}/\;\mu \text{L}$) (r=0.88), hemoglobin (g/dL) and red blood cell count ($x10^{6}/\;\mu \text{L}$) (r=0.87), mean corpuscular volume (fL) and mean corpuscular hemoglobin (pg) (r=0.86), monocytes ($x10^{3}/\;\mu \text{L}$) and leukocytes ($x10^{3}/\;\mu \text{L}$) (r=0.78), red blood cell distribution width - coefficient of variation (%) and red blood cell distribution standard deviation (fL) (r=0.78), monocytes ($x10^{3}/\;\mu \text{L}$) and lymphocytes ($x10^{3}/\;\mu \text{L}$) (r=0.73), granulocytes (%) and granulocytes ($x10^{3}/\;\mu \text{L}$) (r=0.72), and neutrophils (%) and lymphocytes (%) (r=-0.95). For the selection criterion between the pair of variables, the variable most closely correlated with the rest was eliminated. Therefore, erythroblasts (%), mean platelets volume (fL), platelets distribution width (fL), hematocrit (%), red blood cell count ($x10^{6}/\;\mu \text{L}$), eosinophils (%), mean corpuscular volume (fL), red blood cell distribution width - coefficient of variation (%), monocytes ($x10^{3}/\;\mu \text{L}$), granulocytes (%), and lymphocytes (%) were excluded.

### 2.4 Model development

In order to train and evaluate the performance of each ML algorithm in combination with a scaler. Adaboost [70] and bagging [71], gaussian naïve bayes (NB) [72], singular vector machines (SVM) [73], multilayer perceptron (MLP) [74], LR [75], decision tree [76], K-Neighbors [77], and gaussian process (GP) [78] were used during the experimentation phase. Ensemble methods and bagging techniques were used to evaluate and compare the performance of each explainable algorithm against more complex models.

#### 2.4.1 Explainability of the model

In this study, we interpret the coefficients of LR as odds ratios, assuming there is a linear relationship between the model's coefficients and the log-odds (also known as logit) [79]. The logit *ℓ* of the dependent variable is defined as Eq. (1) where $b_{0}$ is the interceptor parameter, $b_{i}$ and $X_{i}$ are the coefficient and the value of each independent variable in the model respectively.

(1) $$\ell =b_{0}+b_{1}X_{1}+b_{2}X_{2}+...+b_{i}X_{i}$$

This interpretation allows us to quantify the effect of each independent variable on the probability of the binary outcome (severe or non-severe) as shown in Eq. (2) where *p* is the probability of prediction to be 1 (severe patient).

(2) $$\ell =\log\left(\frac{p}{1-p}\right)$$

Furthermore, to enhance the interpretability of the coefficients, logits are converted to probabilities instead of being treated on a logarithmic scale. Therefore, Eq. (3) can be obtained by exponentiating and solving Eq. ((1) and (2)).

(3) $$p=\frac{odds}{1+odds}$$

### 2.5 Model evaluation

The models were evaluated using stratified cross-validation with k=5 and 80/20 splits for the training and test sets, with approximately 7,075 and 1,769 blood tests respectively. On each iteration of the stratified cross-validation, the combination of algorithms and scalers was applied in order to find the algorithm and scaler with the best performance for the population study. Accuracy, sensitivity, and specificity were employed as evaluation metrics for the selection of the optimal algorithm.

Upon identifying the best-performing model and scaler combination, model hyperparameters will be fine-tuned to optimise the training of the model. The average of sensitivity and accuracy were weighted to minimise the effect of false negatives while increasing the models' sensitivity because it is more dangerous to falsely classify a high-risk patient as a non-high-risk patient than vice versa.

Finally, events-per-variable (EPV) [80] to assess the adequacy of the dataset in relation to the number of events (positive cases) for each independent variable were included in the model. Furthermore, the standard error and confidence interval of the coefficients are also provided to assess the uncertainty associated with the estimates of the LR coefficients.

## 3 Results

### 3.1 Data description

The COVID-19 cohort of the study included 2,935 patients who tested positive for COVID-19 between 27 February 2020 and 15 April 2021. Of these, 2007 (68%) were outpatients, 394 (13%) and 142 (5%) were admitted to the hospital and the ICU respectively, while 392 (13%) died. After identifying and characterising this cohort, 8844 biochemical and haematological parameters were analysed. Table 2 shows the descriptive analysis of the population on the variable age, days from COVID-19 diagnosis to laboratory test results, as well as the average value of the most frequent tests. It is important to note that days from diagnosis to test refers to the difference in the number of days on which samples are collected.

**Table 2:** Baseline laboratory marker values through test from patients affected with COVID-19 Disease. LDH and GPT stand for lactate dehydrogenase and glutamic pyruvic transaminase respectively.

	Non severe patient test (n = 7240)		Severe patients test (n = 1604)		all patients test (n = 8844)	
Variable	Mean	Std	Mean	Std	Mean	Std

Age	61.43	17.4	75.76	14.26	64.03	17.75
Days from diagnosis to laboratory test result	3.7	4.05	3.19	3.83	3.61	4.01
Glucose (mg/dL)	125.61	57.07	140.3	59.4	128.27	57.77
Creatinine (mg/dL)	0.96	0.83	1.32	1.22	1.03	0.92
Sodium (mEq/L)	138.34	3.65	139.76	6.85	138.6	4.44
Potassium (mEq/L)	4.14	0.52	4.13	0.64	4.14	0.54
Chlorine (mEq/L)	101.91	3.97	102.47	6.91	102.01	4.65
GPT (U/L)	44.19	55.81	39.69	63.01	43.37	57.2
LDH (U/L)	266.45	101.7	387.37	266.46	288.38	153.32
Lipemic index	6.75	8.66	5.06	6.96	6.44	8.4
Glomerular filtration (CKD-EPI) (mL/min)	85.57	25.84	65.7	29.75	81.97	27.67
Icteric index (mg/dL)	0.69	0.32	0.72	0.63	0.69	0.4
C-reactive protein (mg/L)	49.19	58.71	98.13	87.8	58.07	67.63
Hemoglobin (g/dL)	13.26	1.76	12.74	2.12	13.16	1.84
Mean corpuscular hemoglobin concentration (g/dL)	33.35	1.24	32.85	1.4	33.26	1.29
Red Blood Cell Distribution standard deviation (fL)	43.13	4.87	47.19	6.02	43.87	5.33
Hemolysis index	10.81	19.73	12.69	22.24	11.15	20.22
Erythroblasts XT	0.03	0.14	0.09	0.41	0.04	0.22
Leukocytes ($x10^{3}/\;\mu \text{L}$)	7.84	13.35	9.41	8.1	8.12	12.57
Neutrophils ($x10^{3}/\;\mu \text{L}$)	5.48	3.25	7.33	4.61	5.82	3.61
Eosinophils ($x10^{3}/\;\mu \text{L}$)	0.04	0.08	0.02	0.09	0.03	0.08
Granulocytes ($x10^{3}/\;\mu \text{L}$)	0.13	0.32	0.13	0.25	0.13	0.31
Basophiles ($x10^{3}/\;\mu \text{L}$)	0.02	0.02	0.02	0.02	0.02	0.02
Platelets ($x10^{3}/\;\mu \text{L}$)	262.01	114.02	217.24	103.87	253.89	113.56
Monocytes (%)	8.2	3.83	7.02	5.76	7.98	4.27
Basophiles (%)	0.31	0.26	0.23	0.24	0.3	0.26
Neutrophils (%)	70.33	13.72	78.72	14.59	71.85	14.25
Platelets large cell ratio (%)	30.69	8.05	33.72	8.51	31.24	8.22

### 3.2 Results of machine learning models

As detailed in the materials and methods section a stratified cross-validation with k=5 with an 80/20 partition was applied to the study dataset. Table 3 shows the average accuracy values of the stratified cross-validation iterations for each combination of algorithm and scaler. 2% was the average performance difference between the majority of algorithms with the exception of the NB and decision tree whose performance was around 5% worse in terms of average accuracy. Additionally, there is a high degree of similarity in sensitivity and specificity between algorithms, except NB and decision tree, which is in line with the trends observed in the accuracy measure presented in Table 3. Therefore, considering the simplicity and higher interpretability of the LR algorithm in contrast to the other algorithms, its ulterior selection is justified. The LR model showed average values of accuracy (86.53%), specificity (96.38%), precision (69.09%), sensitivity (36.59%), F1 score (38.74%), and AUC (0.8418).

**Table 3:** Average accuracy results of the stratified cross validation iterations for the different ML algorithms and scalers. The best result for each algorithm is shown in bold.

	SVM	LR	K-Neighbors	DecisionTree	NB	RandomForest	MLP	GP	AdaBoost	Bagging
MinMaxScaler	**0.8675**	0.852	0.8632	0.8164	**0.8147**	**0.873**	0.841	0.8524	0.8521	0.8477
StandardScaler	0.8445	**0.8653**	**0.8651**	0.8124	**0.8147**	0.8703	0.8579	0.8515	0.8523	**0.8693**
MaxAbsScaler	0.8633	0.8492	0.8641	0.816	**0.8147**	0.8712	0.8415	0.85	0.8521	0.839
RobustScaler	0.8437	0.8553	0.8593	**0.8177**	**0.8147**	0.8699	**0.8597**	0.8508	0.8521	0.8467
Quant-Normal	0.8576	0.855	0.8549	0.8169	0.8034	0.8712	0.8587	0.8549	0.8523	0.8632
Quant-Uniform	0.8506	0.8512	0.8646	0.8145	0.7844	0.8712	0.8488	0.856	0.8523	0.8677
PowerTransf-yeoJhonson	0.8593	0.8557	0.8637	0.8163	0.801	0.8716	0.8583	**0.8657**	**0.8531**	0.8643

### 3.3 Model optimization

On the basis of the results obtained above, the hyperparameters of the best model were tuned by a grid search algorithm to optimise the training of LR. For instance, the regularisation strength was optimised to improve numerical stability and reduce overfitting [81]. Additionally, the class weight parameter was fine-tuned to adjust the weight of the different classes, which proved to be especially useful for handling the unbalanced dataset [82], [83].

**Figure 5:**
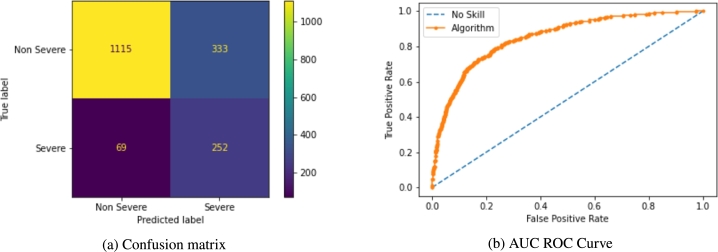
(a) Confusion matrix of logistic regression optimized. Non-severe patients have been defined as COVID-19 non-hospitalized patients while severe patients are those that required intensive care unit care or that died during COVID-19 infection. (b) AUC Roc Curve of logistic regression optimized. The relation of true positive rate and false positive rate is shown comparing logistic regression (solid line) with no-skill algorithm (dashed line).

Model performances were then analysed to assess the achieved improvement after optimization. The optimised LR model, with an EPV [80] of 84.42, showed an accuracy of 77.27%, a specificity of 77%, a precision of 43.07%, a sensitivity of 78,05%, a F1 score of 55.62%, and AUC of 85.12% (Fig. 5a and 5b). Performance metrics comparison of the LR model before and after the optimization step showed a reduction in model accuracy (9.26%), model specificity (19.38%) and model precision (26.02%) along with an improvement of model sensitivity (41.46%), F1-score (16.88%) and AUC (0.94%).

### 3.4 Feature importance

The weight of each of the variables in the LR model was calculated by applying the following Eq. (1). The odds ratio for each prediction was calculated by Eq. ((2) and (3)). This analysis provides an individual and a population-based perspective model explanation. It allowed us to identify which clinical parameters were relevant for predicting COVID-19 disease progression and patient outcomes while facilitating model adoption by providing insights into the internal mechanics of the model without any deep technical knowledge of the mechanisms behind it.

In Table 4 it is shown the most relevant variables to classify patients' outcomes into critically ill or non-critically along with the coefficient, odds ratio, standard error, confidence intervals, and the weight in terms of probability of each relevant variable. Lactate dehydrogenase (LDH), age, red blood cell distribution standard deviation, neutrophils, basophils, and C-reactive protein are the most relevant variables for predicting severe patients' COVID-19 outcomes. On the contrary, platelets, basophils(%), granulocytes, and mean corpuscular haemoglobin concentration were the best indicators that the patient would overcome the infection without requiring admission to the hospital units. It is key to note that these variables do not indicate that they are optimal in themselves for a patient's evolution, but that those patients who did not require hospital admission had certain levels.

**Table 4:** Most influential variables in the severe evolution of patients affected by COVID-19.

Variable	Log odds	Odds ratio	Standard error	Confidence interval (p < 0.001)	Probability of becoming severe
LDH (U/L)	0.821	2.273	0.119	[2.155;2.392]	2.88% (each 10 U/L)
Age	0.663	1.940	0.103	[1.837;2.043]	11.49% (each 10 years old)
Red Blood Cell Distribution standard deviation (fL)	0.338	1.402	0.078	[1.324;1.480	0.82% (each 1 fL)
Neutrophils ($x10^{3}/\;\mu \text{L}$)	0.308	1.361	0.100	[1.261;1.460]	3.82% (each $1x10^{3}/\;\mu \text{L}$)
Basophiles ($x10^{3}/\;\mu \text{L}$)	0.217	1.242	0.123	[1.119;1.365]	5.29% (each $0.01x10^{3}/\;\mu \text{L}$)
C-reactive protein (mg/L)	0.211	1.235	0.046	[1.189;1.280]	1.87% (each 10 mg/L)
Platelets ($x10^{3}/\;\mu \text{L}$)	-0.421	0.656	0.039	[0.617;0.695]	-0.09% (each $1x10^{3}/\;\mu \text{L}$)
Basophiles (%)	-0.276	0.758	0.081	[0.678;0.839]	-43.48% (each 1%)
Granulocytes ($x10^{3}/\;\mu \text{L}$)	-0.233	0.792	0.057	[0.736;0.849]	-47.87% (each $1x10^{3}/\;\mu \text{L}$)
Mean corpuscular hemoglobin concentration (g/dL)	-0.204	0.816	0.050	[0.765;0.866]	-0.53% (each 1 g/dL)

**Figure 6:**
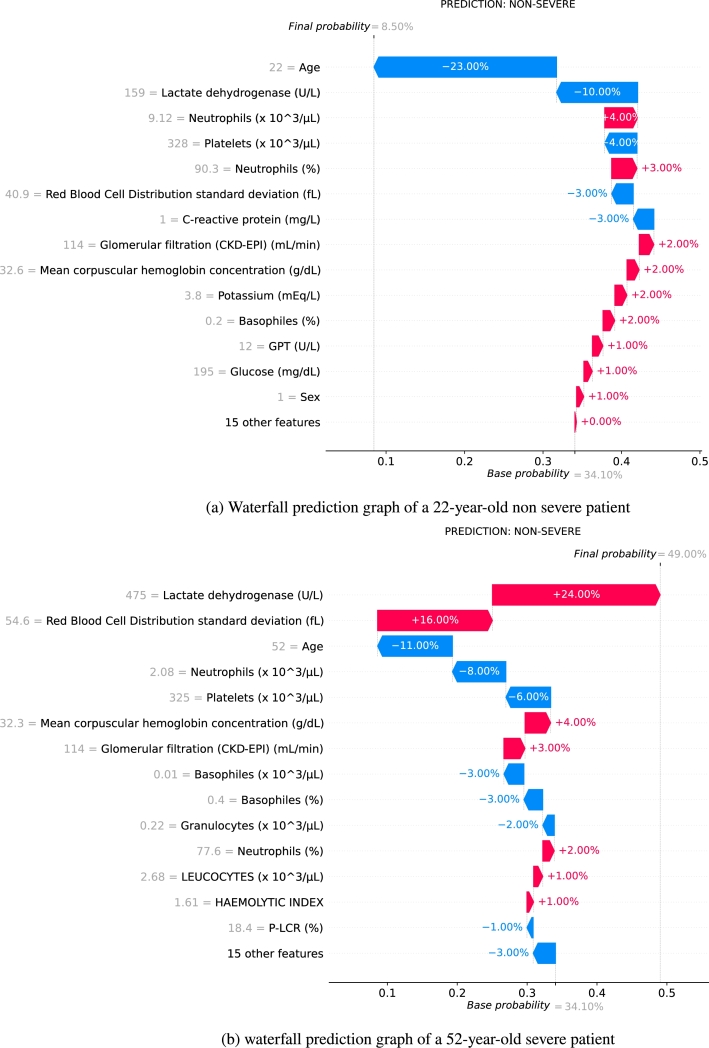
(a) Waterfall with the influence of each variable for the prediction of a 22 year old non severe patient. (b) Waterfall with the influence of each variable for the prediction of a 52 year old severe patient.

Moreover, specific samples in the test dataset were selected to calculate the odds ratio value of each predictor given by Eq. ((2) and (3)) on specific predictions. Fig. 6a and 6b shows which features determined that the patient should be classified as a critically ill patient (red) and which determined that the sample should be classified as a non-critically ill patient (blue).

## 4 Discussion

The aim of this study is to anticipate the likelihood of ICU admission or mortality in patients afflicted with COVID-19 (severe cases) and to ascertain predictive laboratory factors for early diagnosis of patient deterioration. Moreover, it is necessary to highlight the temporality of blood sample collection due to the dynamic changes in the routine blood samples [84]. In this sense, blood samples were collected in 3,6 days on average since the COVID-19 positive and prior to admission, to provide insight into the early conditions of severe patients.

As previously highlighted, AI research on COVID-19 has predominantly relied upon image analysis, comorbidity identification, and laboratory test results [34], [35]. If we analyse the literature concerning the prediction of critically ill patients, different solutions based on CT-Scan images or X-Scan have shown promising potential. In [85], segmented lung slices based on the largest lesion area are used to build a model to predict the severity of patients. Although their proposal has obtained about 1% more accuracy, a 15% improvement in sensitivity has been obtained in our approach. Similarly, authors in [15] show that patients with higher chest CT severity scores have a higher probability of mortality. Even though the identification of the disease progression has been performed consistently well by medical image approaches [34], [86], they are more susceptible in earlier stages of the disease where CT-scans may be normal [37]. Our approach has been developed using blood samples obtained in the early stages of the disease, achieving robust results. Furthermore, it facilitates its application in early diagnosis of a patient's severity since it uses variables that can be obtained in primary care.

Existing AI-based applications for diagnosis using comorbidities have been shown to perform successfully [8], [10], [14], [39], [40]. Although similar results [8], [10], [39] have been obtained, our main contribution is that we extend the scope by identifying patients who will require ICU admission. Regarding [40] we improved the presented results in terms of AUC (11.12%), sensitivity (3.05%) and accuracy (3.27%). Shanbehzadeh et al. [14] used laboratory markers in addition of comorbidities improving accuracy (12.04%) and specificity (11.8%) but results in decreased sensitivity (13.95%) compared to our model. Undoubtedly, although historical data is required to apply comorbidities-based methods, high performance has been achieved by predicting the severity of patients affected by COVID-19. It is important to note that even though this approach allows what-if analysis, expert knowledge is required to maximise the potential of the models. On the contrary, blood sample variables are based on the baseline state of the patient. In any case, the combination of laboratory variables with disease and comorbidities variables could improve the performance, although the complexity of the model would increase.

Moreover, previous studies have explored predictive models based on blood test results and demographic information to predict ICU admission, yielding promising results [21], [87], [88]. Famiglini et al. [87] built LR and decision tree models to predict ICU admission and obtained, on average, an increase of 1.9% in sensitivity compared to us. Meanwhile, we improved specificity and AUC by 5.5% and 3.12%, respectively. In [21], [22], the authors obtain impressive results using laboratory variables, with an average improvement of 18.79% in accuracy, 11.4% in sensitivity, and 9.88% in AUC compared to our model. However, the authors acknowledge the challenge posed by the relatively smaller sample size of ICU patients compared to the non-ICU group, which may have influenced the results. In [12], a histogram-based gradient-boosting which was run with only procalcitonin and ferritin, correctly detected almost all of the COVID-19 patients, both living and deceased (precision > 0.98, recall > 0.98) and determining the lethal-risk levels of procalcitonin and ferritin. In addition, our model obtains competitive results compared to those presented by Pasic et al. [88]. More specifically, even though our model does not include comorbidities, we have improved specificity by 24.1%, while underperforming by 46.13% in precision, 9.95% in sensitivity, and 4.23% in accuracy. Finally, in comparison with previous studies, our model not only predicts patients requiring ICU admission but also identifies the risk of mortality. This extended capability adds significant value to our model in clinical decision-making, aiding in achieving more effective and timely interventions for high-risk patients. Moreover, the robust performance achieved by our model, even when considering only blood laboratory variables, underscores its potential as a practical and efficient solution to address the prediction of deterioration in COVID-19 patients.

Our results are in line with partial results obtained by previous studies, as the variables identified in our research correspond to subgroups reported across different studies in the literature. Specifically, our findings on LDH, age, red blood cell distribution standard deviation, neutrophils, basophils, and C-reactive protein for severe and non-severe COVID-19 cases are supported by the results obtained across various prior investigations [48], [49], [87], [88], [89]. However, the difference in weight across the variables could be due to variations among different populations or settings [90]. It is also important to note that previous studies have reported higher values for the following parameters in patients with greater severity compared to those with mild symptoms: alanine transaminase, aspartate aminotransferase, creatine kinase-MB, gamma-glutamyl transferase, alkaline phosphatase, direct bilirubin, creatine kinase, magnesium, total bilirubin, C-reactive protein, erythrocyte sedimentation rate, international normalized ratio, prothrombin time, D-dimer, ferritin, fibrinogen, procalcitonin, troponin immunological values [22] and lymphocytes [19]. Additionally, cholesterol, high-density lipoprotein cholesterol, triglycerides, amylase, and alkaline phosphatase showed differences in diagnostic evaluation [9], while procalcitonin, D-dimer, erythrocyte sedimentation rate, direct bilirubin, ferritin, absolute count value of neutrophil and lymphocyte were significant factors in assessing mortality [12], [14], [89]. Therefore, the inclusion of these variables could improve the results presented in this study.

Finally, the main limitations of the study are that despite using a larger patient cohort than other studies, the data were obtained from a geographical region of Spain, whose population may be different from other populations around the globe [90]. In addition, although stratified cross-validation was applied, external validation should be performed to strengthen the evidence and generalizability of the model. It is also worth mentioning the variability of levels of blood samples, which could be included to improve the results shown.

Despite these limitations, the current study demonstrates the applicability of AI techniques in the field of health. In addition, (1) we have delved into questions of explainability and interpretation of results with the aim of offering a tool to draw conclusions from what the model has learned. (2), we included a prediction explanation not only to give a prediction but to show the different variables and how they have influenced a specific prediction in order to offer better support for decision-making. (3) We used a reduced number of variables and only required laboratory markers obtained from routine blood tests without requiring database analysis, the patient's age, and sex.

## 5 Conclusions and future works

In this study, we assess the use of laboratory markers with the aim of identifying which patients will require intensive care or suffer from some of the most serious conditions caused by COVID-19, using data from the first positive until before epidemiological discharge or the patient is admitted to ICU. Therefore, 8,844 routine blood tests and demographic variables such as the age and sex of the patients were used to build the model. LR was the best-performing model with an accuracy, sensitivity, and AUC of 77.27%, 78.55% and 0.86 respectively. LDH, age, red blood cell distribution standard deviation, neutrophils and platelets were shown to be the major variables for early diagnosis of the severity of COVID-19-affected patients. Furthermore, we also provide the feature explanation for each prediction to facilitate decision-making by specialists and to enable external validation of the model.

As future work, we aim to conduct external validation of the LR model using data from different hospitals and different periods. Furthermore, the results could be complemented with additional variables, such as clinical images, to gather more comprehensive information about the patient's baseline status. To overcome this issue, one potential solution is to employ another model that combines the diagnoses obtained from both models. Additional variables, such as the effect of ferritin, immunological parameter levels, or biomarkers, could be included to enhance the prediction of severe patient outcomes. Moreover, we intend to incorporate survival analysis in future studies by employing the Cox model and considering survival or admission times which can provide a more comprehensive understanding of the underlying patterns and relationships between variables.

## Ethics declarations

This study was reviewed and approved by the Medicaments Research Ethics Committee of the University and Polytechnic La Fe of Valencia, with the approval number: #2020-181-1. Informed consent was not required for this study because the legitimacy for the processing of personal data based on the anonymised or pseudonymised processing of data without consent under the terms provided in Article 16.3 of Law 41/2002, of 14 November, the basic law regulating patient autonomy and rights and obligations regarding clinical information and documentation, in relation to the second paragraph of the seventeenth additional provision on the processing of health data of Organic Law 3/2018, of 5 December, on the Protection of Personal Data and guarantee of digital rights.

## CRediT authorship contribution statement

**A. Reina-Reina:** Writing – review & editing, Writing – original draft, Visualization, Validation, Software, Methodology, Formal analysis, Data curation, Conceptualization. **J.M. Barrera:** Writing – original draft, Validation, Software, Methodology, Formal analysis, Data curation, Conceptualization. **A. Maté:** Writing – review & editing, Software, Project administration, Methodology, Conceptualization. **J.C. Trujillo:** Writing – review & editing, Software, Resources, Project administration, Methodology, Conceptualization. **B. Valdivieso:** Writing – review & editing, Validation, Methodology, Formal analysis. **María-Eugenia Gas:** Writing – review & editing, Validation, Methodology, Formal analysis.

## Declaration of Competing Interest

The authors declare that they have no known competing financial interests or personal relationships that could have appeared to influence the work reported in this paper.

## Data Availability

The data that support the findings of this study are available from the Medical Research Institute of Hospital La Fe, but restrictions apply to the availability of these data, due to the nature of data which were used after signing a data processing agreement that complies with the requirements of the current legal framework in relation to data processing for the current study, and so are not publicly available. Data pseudo-anonymised are however available from the Medical Research Institute of Hospital La Fe upon reasonable request to any researcher wishing to use them for non-commercial purposes and who could guarantee and demonstrate compliance with national and European legal requirements regarding data protection. Researchers who wish to obtain a copy of the data submit their request to valdivieso_ber@gva.es.
